# Isoflavone supplementation, via red clover hay, alters the rumen microbial community and promotes weight gain of steers grazing mixed grass pastures

**DOI:** 10.1371/journal.pone.0229200

**Published:** 2020-03-13

**Authors:** Brittany E. Harlow, Michael D. Flythe, Isabelle A. Kagan, Jack P. Goodman, James L. Klotz, Glen E. Aiken

**Affiliations:** 1 United States Department of Agriculture, Forage Animal Production Research Unit, Agricultural Research Service, Lexington, Kentucky, United States of America; 2 Department of Plant and Soil Sciences, University of Kentucky, Lexington, Kentucky, United States of America; United States Department of Agriculture, Agricultural Research Service, UNITED STATES

## Abstract

Biochanin A, an isoflavone present in the pasture legume red clover (*Trifloium pratense* L.), alters fermentation in the rumen of cattle and other ruminants. Biochanin A inhibits hyper-ammonia-producing bacteria and promotes cellulolytic bacteria and fiber catalysis *in vitro* and *ex vivo*. Consequently, biochanin A supplementation improves weight gain in grazing steers. Red clover contains biologically active isoflavones that may act synergistically. Therefore, the objective was to evaluate the effect of two levels of red clover hay on growth performance and the microbial community in growing steers grazing mixed grass pastures. A grazing experiment was conducted over 2 early growing seasons (2016 and 2017) with 36 cross-bred steers and twelve rumen-fistulated, growing Holstein steers for evaluation of average daily gain and rumen microbiota, respectively. Steers were blocked by body weight and assigned to pastures with one of four treatments: 1) pasture only, 2) pasture + dry distillers’ grains (DDG), 3) pasture + DDG + low level of red clover hay (~15% red clover diet), or 4) pasture + DDG + high level of red clover hay (~30% red clover diet). DDG were added to treatments to meet protein requirements and to balance total protein supplementation between treatments. All supplementation strategies (DDG ± red clover hay) increased average daily gains in comparison to pasture-only controls (*P* < 0.05), with a low level of red clover supplementation being the most effective (+0.17 kg d^-1^ > DDG only controls; *P* < 0.05). Similarly, hyper-ammonia-producing bacteria inhibition (10–100-fold; *P* < 0.05), fiber catalysis (+10–25%; *P* < 0.05) and short chain fatty acid concentrations were greatest with the low red clover supplement (+~25%; *P* < 0.05). These results provide evidence that lower levels or red clover supplementation may be optimal for maximizing overall microbial community function and animal performance in grazing steers.

## Introduction

Dietary protein supplementation is often used to enhance the nutritional status and overall performance of cattle in grazing systems. Grazing cattle require rumen degradable protein to maximize the ability of their rumen microbiota to catabolize cellulose, but excess ammonia is lost [1]. Excess loss of ammonia is predominately caused by the activity of hyper ammonia-producing bacteria (HAB), which break down incoming amino acids and convert them into ammonia [2]. Although the animal can utilize microbial protein to meet their requirements at maintenance, animals with increased nutritional requirements for growth benefit from rumen -undegraded protein. There are several methods for increasing rumen-undegraded protein including feed processing (*e*.*g*., heat, pressure) and the use of antimicrobials (*e*.*g*., ionophores) [3]. Despite the benefits, there is increasing social and legal pressure to abandon growth promoters that are categorized as antibiotics (*i*.*e*., an antimicrobial of microbial origin). However, antimicrobial growth promoters, and the nitrogen efficiency they provide, are not simply a cost saving measure for producers. Nitrogen brought onto a farm must be captured in plant or animal tissue or it becomes an environmental pollutant, and the reduction in nitrogen pollution by ionophores has been measured and modeled [4]. Thus, growth promotion and feed efficiency are matters of sustainability and environmental stewardship.

The forage legume, red clover (*Trifolium pratense*), contains an isoflavone, biochanin A, that has been shown to inhibit HAB [5–7] and promote cellulolytic bacteria from the rumen [8, 9]. These results suggest a narrow and useful spectrum of activity. Cattle evolved as herbivores consuming diets rich in cellulose and hemicellulose. The enzymes required for fiber degradation are strictly microbial; therefore, cellulolytic bacteria that produce these enzymes are arguably the most important bacteria in the rumen of grazing cattle. It is unlikely that there would be any benefit to inhibiting HAB in grazing animals if cellulolytic bacteria were also inhibited.

Purified biochanin A was used in the earlier experiments that showed effects on HAB and cellulolytic bacteria. A feeding trial (supplemented pasture) with biochanin A verified that the isoflavone promoted average daily gain on supplemented pasture [7]. This latter experiment connected the mechanistic effects of the compound based on the *in vitro* work to the predicted animal growth effect *in vivo*. However, there are two shortcomings to the biochanin A supplemented pasture experiment: 1) chemically purified or synthesized biochanin A is too expensive to use commercially, and 2) using a single purified chemical ignores the total chemical complexity of *T*. *pratense*, including possible synergistic activity with other isoflavones.

Biochanin A is one of the most abundant isoflavones in red cover when its glycosides (an *O*-glucoside, called sissotrin, and an *O*-malonylglucoside) are included [10]. Formononetin did not inhibit HAB in pure culture but was present in “Kenland” red clover tissue in greater concentrations than biochanin A [5]. Relative amounts of biochanin A and formononetin can depend on plant part or maturity [11]. The use of intact red clover tissue is more holistic than the use of purified biochanin A because red clover tissue includes formononetin, biochanin A, the less abundant isoflavones (genistein and daidzein; [12]), the respective O-glucosides (ononin, sissotrin, genistin, and daidzin), and the corresponding malonylglucosides in the complex chemical and physical matrix in which the animal and its microbiota naturally encounter the phytochemicals. In the same regard, red clover hay is a more realistic choice for cattle managers to deliver isoflavones to animals. The advantage of hay over grazed red clover was experimental; *i*.*e*., intake could be controlled. The following experiment was initiated to determine the microbiological and animal growth effects of two levels of supplemental red clover hay on grazing steers. Over two spring grazing seasons, Angus cross steers were supplemented with two levels of red clover hay on endophyte-free cool season grass pasture. A rumen-fistulated Holstein steer was included in each treatment as a microbiological tester. These testers were sampled to enumerate bacteria and obtain microbiota for *ex vivo* digestions. Bacterial culture methods were utilized in the current study as the quantification of specific nutrient-utilizing guilds were of interest and cannot be evaluated with phylogeny-based molecular microbial profiling methods. The hypotheses were that red clover hay supplementation would 1) increase average daily gain in a dose-dependent manner; 2) suppress HAB; 3) promote cellulolytic bacteria and fiber degradation. Fructan-utilizing bacteria were also enumerated as a first attempt to determine potential effects of isoflavones on the rumen microbiota relying on water-soluble carbohydrates in cool season grasses.

## Materials and methods

All procedures were approved by the Institutional Animal Care and Use Committee at the University of Kentucky (protocol #: 2016–2338). General housing and care of the animals were consistent with the Guide to Care and Use of Agricultural Animals in Research and Teaching [13].

### Grazing experiment

The study was conducted in the spring of 2016 and 2017 with different steer groups at the University of Kentucky C. Oran Little Research Center, in Woodford County, Kentucky. Twelve 1.0-ha pastures containing 6-yr-old mixtures of endophyte-free tall fescue [*Lolium arundinaceum* (Schreb.) Darbysh], Kentucky bluegrass (*Poa pratense* L.), and orchardgrass (*Dactylis glomerata* L.) were used in the experiment (Table 1). Soil types were either Maury (fine, mixed, semi-active, mesic Typic Paleudalfs) or McAfee (fine, mixed, active, mesic Mollic Hapludalfs) silt loams. The grasses in both years were vegetative for the duration of the trial, and grazing intensities were adequate in limiting excessive accumulation of mature growth.

**Table 1:** Year effects on the forage mass (FM) and nutritive values (CP; NDF; ADF; and true *in vitro* dry matter digestibility, IVTD) for a cool-season grass mixture and percentages of tall fescue (*Lolium arundinaceum*, TF), Kentucky bluegrass (*Poa pretense*, KBG), and orchardgrass (*Dactylis glomerata*, OG) for grazing trials conducted in 2016 (13 May– 22 July) and 2017 (25 April– 30 June).

		Nutritive Values				Botanical Composition				
Year	FM	CP	NDF	ADF	IVTD	TF	OG	KBG	Other	Weeds
	-kg DM ha^-1^-	------------g kg^-1^ DM------------				-----------------% of total-----------------				
2016	3269	95.6	606.1	357.9	626.7	36.4	17.9	43.7	0.6	1.4
2017	2890	136.8	605.7	354.1	680.2	39.9	20.3	38.4	0.9	0.5
SEM	85	1.8	2.6	1.6	2.1	1.3	2.3	2.6	0.2	0.3
*Sig*^a^	***	***	NS	NS	***	NS	NS	NS	NS	NS

^a^ NS, not significant (*P* > 0.05)

***Significant at the 0.001 probability value

Pastures were blocked according to the average slope and soil type and were assigned to 1 of 4 treatments with 3 replications: 1) pasture only (control), 2) pasture + supplementation with dry distillers’ grains (DDG; 2.27 kg DDG steer^-1^), 3) pasture + DDG + low level of red cover (Low RC; 1.51 kg DDG steer^-1^, 0.91 kg RC steer^-1^), or 4) pasture + DDG + high level of red clover (High RC; 0.75 kg DDG steer^-1^ + 1.81 kg RC steer^-1^). The levels of red clover hay (2016: DM basis: 16% CP, 32% ADF, 39% NDF, 74% IVTD; 2017: DM basis: 14% CP, 36% ADF, 46% NDF, 74% IVTD) were chosen to represent a 250 kg steer (assuming intake 2.5% BW) consuming a 15% or 30% red clover diet for the low and high treatments, respectively. The DDG was corn-based with different batches used in each year (Woodford Feed and Fertilizer; Versailles, KY; 2016: DM basis: 25% CP, 19% ADF, 27% NDF, 83% IVTD; 2017: DM basis: 25% CP, 20% ADF, 28% NDF, 80% IVTD) and was fed as an isoflavone-free protein source to ensure that all supplements were balanced for protein content. Cattle in pastures that received supplemental feed were group fed at approximately 0800 h each morning. The supplements were placed in 2.1 m feed bunks to prevent crowding-related feed competition and to ensure that all supplemented steers had immediate access to the feed in the bunks. All supplemental feed offered was consumed by the next feeding (within 24 h) over the course of the study.

### Isoflavone analyses

Prior to the start of the study, the red clover hay was analyzed for isoflavone concentrations (2016: 0.17 mg g^-1^ daidzein equivalents, 0.17 mg g^-1^ genistein equivalents, 2.11 mg g^-1^ formononetin equivalents, 1.00 mg g^-1^ biochanin A equivalents, 3.45 mg g^-1^ total isoflavone aglycone equivalents; 2017: 0.15 mg g^-1^ daidzein equivalents, 0.14 mg g^-1^ genistein equivalents, 4.47 mg g^-1^ formononetin equivalents, 1.63 mg g^-1^ biochanin A equivalents, 6.39 mg g^-1^ total isoflavone aglycone equivalents).

The extraction procedure was the same in both years. Red clover hay was ground through a 1-mm mesh in a Wiley mill. Tissue (100 to 250 mg) was extracted in 85% methanol containing 0.5% aqueous acetic acid, by sonicating 30 min (model 5510 sonicating water bath, Bransonics Corporation, Danbury, CT). Water (3 mL) was added to the extracts, giving a final solvent composition of 60% methanol in 0.35% acetic acid. The mixture was centrifuged 8 min at 2200 × *g* and 25°C. The supernatant was filtered through a 0.45-μm GHP hydrophilic membrane (Pall Corporation, Port Washington, NY).

In 2016, isoflavones were separated by HPLC (model 1100, Agilent, Santa Clara, CA) on a LiChrospher RP-18 endcapped column (250 mm length × 4.6 mm i.d.) at 28°C. The mobile phase consisted of 1.5% acetic acid (solvent A) and methanol (solvent B), and the gradient was that described by Kagan [14]. Detection was at 254 nm. Biochanin A, formononetin, genistein, daidzein, and their respective glucosides (sissotrin, ononin, genistin, and daidzin) were identified by comparing retention times to those of standards purchased from Sigma-Aldrich (St. Louis, MO) or Indofine Chemical (Hillsborough, NJ). Isoflavones were quantified from 7- or 8-point standard curves for the four aglycones. The glucosides were quantified with the standard curves for the corresponding aglycones.

In 2017, isoflavone extracts were analyzed by LC-MS on a Waters Acquity UPLC coupled to a Waters Synapt G2 (q-ToF) high resolution mass spectrometer. Chromatographic separation was obtained using a Waters BEH C18 UPLC column (1.7 μm, 2.1 mm x 150 mm). The mobile phase employed a mixture of water containing 0.1% formic acid (solvent A) and acetonitrile containing 0.1% formic acid (solvent B) in a linear gradient from 20% B to 80% B at a flow rate of 0.35 mL/min. The high-resolution mass spectrometer was operated in positive ion electrospray mode with a resolving power of ~14,000 and scanned from 100 to 1000 Da in 0.3 s. Leucine enkephalin was used to provide a lock mass (m/z 554.2615). Quantification of isoflavones was performed using QuanLynx software with a linear calibration curve and internal standard method, using flavone as the internal standard. Extracted ion chromatograms with a mass window of 0.02 Da around the accurate mass of each analyte were used to calculate peak areas. Because standards of the malonyl-glucosides were not available, one portion of each sample was analyzed as-extracted and a second portion was heated at 85°C for 5 h to hydrolyze isoflavone malonyl-glucosides to their corresponding isoflavone glucosides. Concentrations of biochanin A malonyl-glucoside and formononetin-malonyl-glucoside were determined by difference between hydrolyzed and un-hydrolyzed portions.

### Cattle management and performance

Two types of cattle, 8- to 10-month old Holsteins and cross-bred steers of Angus breeding, were included in this study to make different measurements. The cross-bred Angus steers were used for weight gain measurements. Additionally, a rumen-fistulated Holstein was included with each group to determine the effect of each treatment on rumen microbiological parameters.

Thirty-six cross-bred Angus steers were blocked by body weight (BW) for random assignment to pastures in spring 2016 (13 May to 22 July, 71 days) and 2017 (25 April to 30 June, 67 days) for evaluation of average daily gain. Twelve rumen-fistulated, growing, Holstein steers were randomly assigned to pastures in spring 2016 (9 June to 22 July, 44 days) and 2017 (18 May to 29 June, 43 days) for evaluation of the rumen microbiota. Pastures were continuously stocked with 3 (all Angus, first ~3 wks) steers or 4 steers in each pasture (3 Angus + 1 Holstein, last ~6 wks). Different cross-bred Angus steers were used in each grazing trial with mean initial BW of 237 ± 29 (SD) kg steer^-1^ and 223 ± 12 (SD) kg steer^-1^ in 2016 and 2017, respectively.

The cross-bred Angus steers were weighed on the initial and final days of each year’s grazing trial following a 12- to 14-h fast from water and feed (shrunk BW). Angus steers were placed on the pasture approximately 1-wk prior to obtaining initial shrunk BW to adjust to pastures, and to the daily feeding of experimental diets. Prior to placement on pastures, all steers received an anthelmintic according to manufacturer’s directions (LongRange; eprinomectin, Merial, Inc., Duluth, GA). The steers were not implanted with anabolic steroids. Cattle were provided free choice water and trace minerals (zinc, 3.5 g kg^-1^ DM minimum, manganese, 2.0 g kg^-1^ DM minimum, copper, 0.3 g kg^-1^ DM minimum, selenium, 0.09 g kg^-1^ DM maximum, iodine, 0.07 g kg^-1^ DM minimum, and cobalt, 0.05 g kg^-1^ DM minimum).

### Pasture management and measurements

Pastures were fertilized with urea to provide 67 kg N ha^-1^ for each application (2016: 1 April; 2017: 5 April). Soil phosphorus and potassium were above levels recommended for cool season grass pastures [15]. All pastures were treated with herbicide (Grazon Next, Dow AgroSciences, Indianapolis, IN; 15 April; 2017: 18 April) to provide 117 g ha^-1^ of aminopyralid (2-pyridine carboxylic acid, 2-amino-3,6-dichloropicolinic acid) and 934 g ha^-1^ of 2,4-D [(2-dichlorophenoxy) acetic acid] to kill weeds and all clovers.

Forage mass was measured using a disc meter at the beginning (2016: 16 May; 2017: 2 May), midpoint (2016: 8 June; 2017: 1 June), and end (2016: 20 July; 2017: 28 June) of each grazing trial ([16], except the falling plate was 32 cm in diameter). Disk meter heights were recorded for 50 random locations within each pasture. Calibration samples for estimating total forage mass were collected on 20 May, 14 June, and 20 July in 2016 and 4 May, 6 June, and 28 June in 2017. The calibration samples were collected by clipping forage below the disc meter plate at the surface of the soil at five random locations in each pasture. All samples collected were dried at 60°C for 48 h in a forced air drying oven and subsequently weighed. Calibration equations were determined by regressing sample dry weight over disc meter heights for estimating forage mass (kg DM ha^-1^) from the mean disk meter height recorded for each pasture.

To assess pasture nutrient composition, three 0.25 m^2^ quadrat simples were clipped to a 2.5 cm height at randomly chosen locations in each pasture (2016: 18 May, 8 June, 21 July; 2017: 2 May, 1 June, 28 June). Quadrat samples were then dried as described above, ground to pass through a 2 mm screen using a Wiley mill, and analyzed for CP, ADF, NDF, and true *in vitro* digestible DM (IVTD). Crude protein was analyzed using a Leco FP-215 N Analyzer (CP = % N × 6.25; Leco Corp., St. Joseph, MI). Acid detergent fiber and NDF were analyzed using an ANKOM fiber analyzer as a modification to procedures of Goering and Van Soest [17]. True IVTD was determined by following procedures for estimating true digestibility using the ANKOM Daisy II Incubator [18].

Botanical composition was estimated for each pasture using a point-transect method that used a 50 m transect tape placed on the top of the canopy at six randomly chose sites within each pasture (n = ~200 points pasture^-1^; June in both 2016 and 2017). Herbage of the forage species closest to each point (1.0 m spacing) was recorded to determine an estimated percentage of occurences for each forage species in top aspects of the canopy.

Tall fescue in each pasture had been previously determined to be endophyte-free [7, 19]. To verify that the tall fescue stands were still free of endophyte, single tiller samples (n = 25 randomly chosen from each pasture) were collected to determine ergovaline concentrations. Each tiller was clipped at the crown of each plant, placed on ice and subsequently freeze dried (Botanique Model 18DX48SA freeze drier; Botanique Preservation Co., Peoria, AZ). After freeze drying, samples were finely ground (1 mm screen) and assayed for ergovaline by HPLC with fluorescent detection (modified procedure, [20, 21]). Averaged over all pastures, the ergovaline concentrations were negligible (0.03 ± 0.01 μg g^-1^ DM) and therefore, posed no risk for fescue toxicosis to impact animal performance [22].

### Fistulated holstein sampling

Rumen fisulated Holstein steers were sampled for rumen measurements at 4 and 6 weeks post-initiation of grazing and treatments (2016: 21–23 June and 19–21 July; 2017: 12, 13, 15 June and 27–29 June). Each block of steers (n = 4 steers block^-1^) was sampled on separate days to ensure timely sample collection and processing. On each sample day, rumen contents (~ 1 kg) were collected from each steer individually in an airtight, insulated container. Immediately post-collection, ~250 g of the contents were strained through 3 layers of cheesecloth to collect rumen fluid. A portion of the collected rumen fluid (15 mL) was placed on ice and frozen for later fermentation end-product analyses (described below). Lastly, within 10 min of sample collection, fresh rumen fluid was subjected to 10-fold dilution (w/w) with anaerobic phosphate buffered saline (PBS; pH 7.4, N_2_-sparged; 8 g NaCl, 0.2 g KCl, 1.44 g Na_2_PO_4_, 0.24 g KH_2_PO_4_ per L), mixed until homogenous, and then serially diluted (10-fold w/w, anaerobic PBS) for the inoculation of bacterial enumeration media (described below). Technical replicates were not performed for bacterial enumeration as previous experiments indicated they are unnecessary in obtaining reproducible viable numbers [23]. After all animals from each block were sampled, remaining rumen contents were transported to the laboratory for the determination of rumen fluid pH and used as the inoculum for *ex vivo* rumen digestibility experiments (described below; within 1 h of collecion).

### Media and bacterial enumerations

Two basal media types were used in the experiment, but each was modifed to facilitate the growth or metabolic activity of particular functional groups of rumen microorganisms. Basal medium 1 was based on Stack and colleagues [24]. This medium was buffered to maximize growth and contained SCFA to enhance initial rates of fiber digestion. It contained (per liter): 240 mg KH_2_PO_4_, 240 mg K_2_HPO_4_, 480 mg (NH_4_)_2_SO_4_, 480 mg NaCl, 64 mg CaCl_2_ · 2H_2_O, 100 mg MgSO_4_ · 7H_2_O, 600 mg cysteine hydrochloride, 1,000 mg Trypticase, 500 mg yeast extract, 1 mg phenylacetic acid, 3.1 mL VFA solution (7.15 mol L^-1^ acetic acid,1.92 mol L^-1^ propionic acid, 783.66 mmol L^-1^ butyric acid, 222.28 mmol L^-1^ valeric acid, 216.19 mmol L^-1^ isovaleric acid, 264.13 mmol L^-1^ isobutyric acid, 227.87mmol L^-1^ 2-methylbutyric acid); initial pH 6.7; autoclaved (121°C, 103 kPa, 20 min) to remove O_2_ and cooled under O_2_ –free CO_2_. The buffer (4,000 mg Na_2_CO_3_) was added before dispensing and autoclaving for sterility.

Basal medium 2 was designed for use in select enumeration media preparation (described below). Basal medium 2 contained (per liter): 240 mg KH_2_PO_4_, 240 mg K_2_HPO_4_, 480 mg Na_2_SO_4_, 480 mg NaCl, 64 mg CaCl_2_ · 2H_2_O, 100 mg MgSO_4_ · 7H_2_O, and 600 mg cysteine hydrochloride, with additional vitamins and trace minerals as previously described [24]. The initial pH was adjusted to 6.7 and the medium was autoclaved (121°C, 103 kPa, 20 min) to remove O_2_ and cooled under O_2_-free CO_2_. The buffer (4,000 mg Na_2_CO_3_) was added before dispensing and autoclaving for sterility.

Total cellulolytic bacteria were enumerated in basal medium with Whatman no. 1 filter paper strips as the primary substrate [24]. The inoculated tubes were incubated for 10 d at 39°C, and the final dilution exhibiting bacterial growth (visual examination, filter paper dissolution) was recorded as the viable number of cellulolytic bacteria.

Total fructan-utilizing bacteria were enumerated in basal medium 1 that was modified to exclude phenylacetic acid and other VFA. Inulin (15 mg mL^-1^) from chicory (BENEO Inc, Morris Plaines, NJ) was added as the substrate. The tubes were inoculated and incubated (39°C, 3 d), and the final dilution exhibiting bacterial growth (visual examination, OD_600_) was recorded as the viable number.

Total peptide-utilizing and amino acid-utilizing bacteria were enumerated in basal medium 2 amended to include either 15 mg mL^-1^ Trypticase or 15 mg mL^-1^ Casamino acids, respectively (Becton Dickinson, Franklin Lake, NJ). Inoculated tubes were incubated (39°C, 3 d), and the highest dilution exhibiting bacterial growth (visual examination, OD_600_) was recorded as the viable number.

Total gelatin-hydrolyzing bacteria were enumerated in basal medium 2 amended to include 100 mg mL^-1^ gelatin (added prior to 2^nd^ autoclaving step). Tubes were inoculated and incubated (39°C, 5 d), and the highest dilution exhibiting bacterial growth (visual examination of gelatin hydrolysis after 1 h at 4°C) was recorded as the viable number.

### Mixed rumen microorganism cell suspensions

Collected rumen digesta (0.5 kg) were transported to the laboratory in an airtight, insulated container, within 1 h of collection. Immediately upon arrival to the laboratory, rumen cell suspensions (for each steer individually) were prepared as previous described by [7, 8, 9]. Briefly, rumen digesta samples were squeezed through 3 layers of cheesecloth to remove large plant fibers. The pH was taken of the collected rumen fluid using a pH meter, and the rumen fluid was then subjected to a low-speed centrifugation (200 × *g*, 10 min) to remove remaining small plant particles and protists (Sorvall RC-6; Thermo-Fisher Scientific, Waltham, MA). Supernatants were collected and subjected to high-speed centrifugation (25,654 × *g*, 5 min) to collect planktonic prokaryotes. Residual supernatants were discarded and the remaining pellets were washed by re-suspension in basal medium 1. Again, cells were subjected to high-speed centrifugation (25,654 × *g*, 10 min), supernatants were discarded and pellets were resuspended and transferred into a CO_2_-sparged glass vessel (2 L), and re-suspended in basal medium 1. The optical density of the cell suspensions at (600 nm) was adjusted to ~ 5 with basal medium 1. Light microscopic analyses revealed prokaryote-sized cells with no obvious plant fiber or protists.

### *Ex vivo* rumen digestibility

An experiment was conducted to determine how the treatments in the current study impact *ex vivo* dry matter digestibility (EVDMD) at 6 wks post-treatment initiation. The term *ex vivo* is used in description of this experiment because the treatments were applied to the animals in the field and not in the laboratory, as is characteristic of *in vitro* experiments. Two different inocula were tested to determine if EVDMD differences were dependent on the presence of isoflavones in the rumen fluid. Inoculum 1 was a 1:1 dilution of rumen fluid in basal medium 1, and inoculum 2 consisted of the mixed rumen microorganism cell suspensions (prepared as described above). Prepared inocula were dispensed into anaerobic (CO_2_) serum bottles containing 20 mg mL^-1^ of finely ground (Wiley mill, 2 mm screen) hay (DM basis: 12.8% CP, 36.3% ADF, 56.3% NDF, 21.8% NFC; DE: 2.24 Mcal kg^-1^), DDG (as described above), or corn (DM basis: 10.9% CP, 3.2% ADF, 8.6% NDF, 61.6% Starch, 3.1% WSC, 8.0% ESC; DE: 3.51 Mcal kg^-1^). The bottles were incubated (39°C) for 48 h. Samples were collected via tuberculin syringes at 0 and 48 h for pH and later ammonia analyses (described below). At the end of the incubation period, serum bottle contents were filtered and residual organic matter was collected in Gooch crucibles via vaccuum filtration with sequential washing of the residue with hot distilled water (90°C) and acetone [9]. The collected residues were dried (55°C) in a forced air drying oven and weighed after 48 h, or until a consistent weight was obtained.

        % EVDMD was calculated by using the following equations:

(EmptyCrucibleDryWeight+InitialDrySampleWeight)–Post‐incubationCrucible/SampleDryWeight=DMD

(DMD÷InitialDrySampleWeight)×100=%EVDMD

### Predominant cellulolytic bacteria % EVDMD

In order to determine how the treatments in the study impacted the numerically predominant cellulolytic bacteria in culture fermentative efficiency specifically, the highest total cellulolytic bacteria enumeration dilution was used as an inoculum for the evaluation of DMD at 6 wks post-treatment initiation. Immediately after cellulolytic bacteria viable number was determined (10 d of incubation), predominant cellulolytic bacteria were inoculated (5% v/v) into serum bottles containing cell suspension media and finely ground (2 mm screen) hay (20 mg mL^-1^, described above). The inoculated bottles were incubated for 24 h (39°C) and % EVDMD was determined (as described above).

### Fermentation end-product analyses

Rumen fluid samples were thawed and clarified in a microcentrifuge (21,000 × *g*, 2 min). Short chain fatty acids (lactate, acetate, propionate, butyrate, IVMB: isovalerate and/or methylbutyrate, and valerate) were quantified using a Summit HPLC (Dionex, Sunnyvale, CA) equipped with an anion exchange column (Aminex, HP-87H; Bio-Rad, Hercules, CA), refractive index (Shodex/Showa Denko, Kanagawa, Japan) and UV detector. The column was operated isocratically at 50°C with a 0.4 mL min^-1^ flow rate, a 0.1 mL injection volume, and a H_2_SO_4_ (0.17 N) mobile phase.

Ammonia concentrations in rumen fluid were determined using a colorimetric method ([25], as modified by [26]).

### Statistical analyses

Prior to statistical analyses bacterial enumerations were normalized by log transformation. All data were analyzed using PROC MIXED of SAS, with pastures being used as the experimental unit (SAS version 9.3, SAS Inst. Inc., Cary, NC). In the analyses for the bacterial enumerations, rumen pH and SCFA (measurements taken at 4 and 6 wks), block, time during the grazing season, treatment, year, and their interactions were analyzed as fixed effects and animal as the random variable. In the analyses for the pasture variables, ADG and the *ex vivo* measures (measurements only taken at 6 wks) the same design was used excluding the fixed effect of time during the grazing season. The Kenward-Roger method was used to compute the denominator degrees of freedom for the fixed effects and the repeated statement utilized the compoud symmetry covariance structure. Mean separations were done using the PDIFF option of SAS. Statistical significance was set at *P* < 0.05 with trends observed at *P* < 0.10.

## Results and discussion

### Pasture measures

None of the forage variables were affected by block or treatment and there was no treatment × year interaction on any of the variables (*P* > 0.05); however, some pasture variables differed between 2016 and 2017. Mean forage mass was less in 2016 than in 2017 (*P* < 0.01; Table 1). However, overall forage mass was high in both years, which indicates that the stocking rates utilized did not limit steer performance due to low forage mass [27].

Nutritive values reported are representative of the whole canopy (above 2.5-cm height) and do not reflect what the steers selectively grazed. Both ADF (*P* = 0.17) and NDF (*P* = 0.92) content of the pasture were similar between years. Crude protein pasture concentrations were 35% greater in 2017 than in 2016 (*P* < 0.01). True *in vitro* dry matter digestibility was also higher in 2017 than in 2016 (*P* < 0.01); however, mean IVTD was higher than 600 g kg^-1^ DM and therefore high over both years.

Overall, botanical composition of the pastures remained consistent between 2016 and 2017 (*P* > 0.10). Kentucky blue grass (~41%) and tall fescue (~38%) were predominant in pastures. Orchard grass accounted for ~19% of pasture composition with a negligible amount of other grasses and weeds present (< 1.5%, in both cases). Herbicide was applied in early April both years to kill both weeds and all clovers and likely contributed to the botanical compositions observed.

### Steer performance

**Fig 1:**
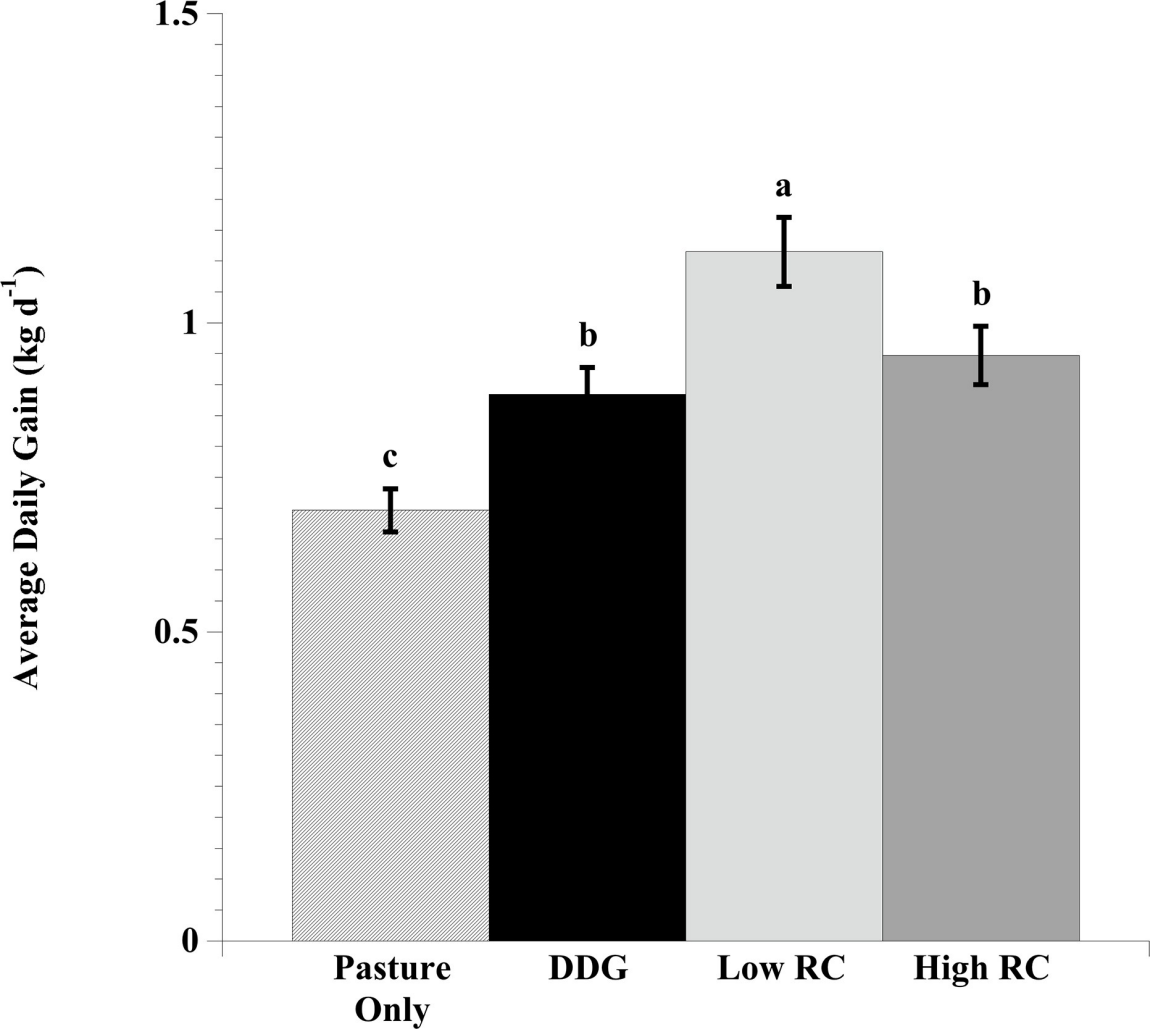
Effect of dried distiller’s grains (DDG) with or without red clover hay (RC) compared with pasture-only control on average daily gain (mean ± SEM bars) of steers grazing a mixture of endophyte-free tall fescue (*Lolium arundinaceum*), Kentucky bluegrass (*Poa pratensis*), and orchardgrass (*Dactylis glomerata*) during the early season (2016: 13 May to 22 July, 71 d; 2017: 25 April to 30 June, 67 d). Thirty-six cross-bred Angus steers were blocked by body weight (BW; 12 pastures, n = 3 steers pasture^-1^) for random assignment to 1 of 4 treatments: Pasture only control, DDG (pasture + 2.27 kg DDG steer^-1^), Low RC (pasture + 1.51 kg DDG steer^-1^ + 0.91 kg RC steer^-1^), or High RC (pasture + 0.75 kg DDG steer^-1^ + 1.81 kg RC steer^-1^). The steers were weighed on the initial and final days of each year’s grazing trial following a 12- to 14-h fast from water and feed. Steers were placed on the pasture approximately 1-wk prior to obtaining initial shrunk BW. Means lacking a common letter are different (*P* < 0.05); Year: *P* < 0.01, Treatment: *P* < 0.01, Year × Treatment: *P* = 0.54.

Steer ADG was affected by supplement treatment (*P* < 0.01) and year (*P* < 0.01); however, no treatment × year interaction (*P* = 0.54) was observed (Fig 1). The ADG recorded overall in 2016 was lower than in 2017 (*P* < 0.05). This year difference could be attributed to higher CP and overall IVTD pasture nutritive values in 2017. Additionally, higher ADG was observed in steers supplemented with DDG ± red clover hay in comparison to the pasture-only controls (*P* < 0.05). As previous discussed, CP from the pasture alone was marginal in meeting the CP requirements of growth in both years, therefore, additional protein supplementation could provide animal performance benefits under CP limiting conditions [28].

Steers supplemented with DDG and a low level of red clover hay (0.91 kg RC steer^-1^) had greater ADG (1.12 kg d^-1^) than steers supplemented with DDG alone (0.88 kg d^-1^) or a high level of red clover hay (1.81 kg RC steer^—1^; 0.95 kg d^-1^; *P* < 0.05). In the current study, steers supplemented with DDG and a high level of red clover hay had statistically similar weight gains to steers supplemented with DDG only (*P* > 0.05), but gains were higher numerically (+0.07 kg d^-1^).

The results of the current study are consistent with a previous study that demonstrated a tendency for increased ADG with the addition of the purified red clover isoflavone biochanin A to DDG supplemented pasture over protein-matched DDG-only controls [7]. In the aforementioned study, growing steers were supplemented with 6.5 g of biochanin A per head daily to represent expected consumption of this isoflavone with a 30% red clover diet [5]. In contrast, steers in the current study were consuming 0.9–1.4 g or 1.8–3.0 g of biochanin A equivalents per head daily on the low and high levels of red clover hay supplementation, respectively. However, total isoflavones in the high level of supplemented red clover were comparable (6.2–11.6 g total isoflavones d^-1^) and similar gains were observed with the treatments in both studies designed to simulate a 30% red clover diet ([7]: 0.93 kg d^-1^, vs. current study: 0.95 kg d^-1^). These results indicate that the benefits of red clover on growth performance is not dictated by biochanin A concentrations alone, but also by the concentrations of other isoflavones. Additionally, this study demonstrated that a low level of red clover supplementation was most effective at improving weight gain in growing steers (+0.17 kg d^-1^) indicating that there is an optimum level of isoflavone supplementation for physiological benefits and that higher levels may be ineffective or less effective in improving animal performance. Future research is required to evaluate lower levels of red clover isoflavone supplementation and their ability to improve performance of growing cattle.

### Rumen metabolites

Steers supplemented with DDG ± red clover hay had greater concentrations of acetate (*P* < 0.01), propionate (*P* < 0.01), butyrate (*P* < 0.01), and total SCFA (*P* < 0.01) than pasture-only controls (Table 2). Although, steers supplemented with DDG only and high red clover had similar levels of SCFA (*P* > 0.05, in all cases), low red clover supplemented steers had the greatest concentrations of acetate (+ 22%), propionate (+ 24%), butyrate (+ 26%) and total SCFA (+ 23%, in comparison to pasture-only controls; *P* < 0.05, in all cases). No lactic acid was detected in any rumen sample, and no treatment differences were observed for branched VFA (isovalerate or 2-methyl butyrate; *P* = 0.08) or valerate (*P* = 0.18) concentrations.

**Table 2:** Effect of dried distiller’s grains (DDG) with or without red clover hay (RC) compared with a pasture-only control on rumen short chain fatty acids (SCFA) and pH.

SCFA (mM)	Treatments^1^				Statistics^2^	
	Pasture-Only	DDG	Low RC	High RC	Sig	SEM
**Acetate**	32.5^c^	40.3^b^	50.0^a^	41.6^b^	*P* < 0.01	0.90
**Propionate**	14.5^c^	18.5^b^	22.6^a^	17.9^b^	*P* < 0.01	0.69
**Butyrate**	6.4^c^	8.2^b^	10.4^a^	8.3^b^	*P* < 0.01	0.22
**IVMB**	1.4	1.7	1.8	1.5	*P* = 0.08	0.10
**Valerate**	0.9	1.1	1.1	1.0	*P* = 0.18	0.06
**Total SCFA**^3^	53.4^c^	66.9^b^	83.0^a^	67.8^b^	*P* < 0.01	1.56
**Rumen pH**	6.60^a^	6.24^c^	6.30^b^	6.23^c^	*P* < 0.01	0.01

^1^Pasture-only: control; DDG: Pasture + 2.27 kg DDG steer^-1^; Low RC: Pasture + 1.51 kg DDG steer^-1^ + 0.91 kg RC steer^-1^; High RC: Pasture + 0.75 kg DDG steer^-1^ + 1.81 kg RC steer^-1^

^2^ Means lacking a common letter are different (*P* < 0.05)

^3^Total SCFA: Acetate + Propionate + Butyrate

Supplementation, regardless of treatment, decreased rumen pH in comparison to pasture-only controls (*P* < 0.01). Despite an increase in SCFA concentration in the rumen with low-level red clover hay supplementation, rumen pH was higher than steers supplemented with DDG only or a high-level of red clover hay (*P* < 0.05).

### Rumen microbiota

When steers consumed pasture, 10^7^–10^9^ and 10^9^–10^10^ cells mL^-1^ total amino acid-utilizing and peptide-utilizing bacteria were observed, respectively (Table 3). There were year (*P* < 0.01) and treatment (*P* < 0.01) effects for total amino acid-utilizing bacteria, but no effect of time or any interactions between these variables (*P* > 0.05). Overall, fewer amino acid-utilizing bacteria were detected in 2017 (1.6 × 10^8^ cells mL^-1^) than in 2016 (3.4 × 10^8^ cells mL^-1^; *P* < 0.05). Protein supplementation with DDG alone increased the viable number of amino acid-utilizing bacteria (*P* < 0.05). In contrast, when red clover hay was provided amino acid-utilizing bacteria were inhibited (10–100-fold), with the low level of supplementation being most effective (*P* < 0.05).

**Table 3:** Effect of dried distiller’s grains (DDG) with or without red clover hay (RC) compared with a pasture-only control on the viable number of rumen bacterial guilds.

Bacterial Enumerations (cells mL^-1^)	Treatments^1^				Statistics^2^	
	Pasture-Only	DDG	Low RC	High RC	Sig	SEM (log transformed)
**Amino Acid-Utilizing**	3.78×10^8b^	5.50×10^8a^	3.03×10^7d^	4.68×10^7c^	*P* < 0.01	0.09
**Peptide-Utilizing**	4.75×10^9a^	4.00×10^10b^	1.90×10^9c^	1.38×10^9c^	*P* < 0.01	0.18
**Gelatin-Utilizing**	9.54×10^7^	3.44×10^8^	1.10×10^8^	1.79×10^8^	*P* = 0.33	0.24
**Fructan-Utilizing**	5.50×10^10a^	1.53×10^10b^	1.68×10^9c^	2.95×10^9c^	*P* < 0.01	0.14
**Cellulolytic**	1.30×10^9^	4.75×10^8^	1.45×10^9^	1.11×10^10^	*P* = 0.45	0.25

^1^Pasture-only: control; DDG: Pasture + 2.27 kg DDG steer^-1^; Low RC: Pasture + 1.51 kg DDG steer^-1^ + 0.91 kg RC steer^-1^; High RC: Pasture + 0.75 kg DDG steer^-1^ + 1.81 kg RC steer^-1^

^2^ Means lacking a common letter are different (*P* < 0.05)

Similar results were observed for total peptide-utilizing bacteria enumerations (Table 3). There were year (*P* < 0.01), treatment (*P* < 0.01), and time (*P* < 0.01) effects for peptide-utilizing bacteria, but no interactions between the aforementioned variables (*P* > 0.05). Overall, fewer peptide-utilizing bacteria were detected in 2017 (3.3 × 10^9^ cells mL^-1^) than in 2016 (2.1 × 10^10^ cells mL^-1^) and the viable number detected decreased from wk 4 to wk 6 of grazing (*P* < 0.05). Supplementation with DDG increased peptide-utilizing bacteria ~10-fold in comparison to pasture-only controls (*P* < 0.05). An increase in HAB in the DDG treatment was expected because of the protein content of the DDG (~25%). It follows that amino acid- and peptide-utilizing bacteria would be more numerous when protein is more available. Red clover hay supplementation inhibited peptide-utilizing bacteria, regardless of the level provided (*P* < 0.05). Despite differences observed in both amino acid and peptide-utilizing bacteria, total gelatin-utilizing bacteria (proteolytic bacteria; range: 1 × 10^5^–1 × 10^9^ cells mL^-1^, avg: 1.82 × 10^8^ cells mL^-1^) were unaffected by treatment (Table 3; *P* = 0.33). This latter result supports the long-standing hypothesis that proteolysis and amino acid fermentation are distinct metabolic steps in the rumen because they are carried out by ecologically distinct groups [25].

The hypothesis that HAB would be inhibited by biochanin A in the hay was supported by the results. HAB, including both amino acid- and peptide-fermenting bacteria, were less numerous in the treatments that included red clover hay. These results were consistent with previous studies in which biochanin A inhibited HAB [5–7, 14]. Recent results by Melchior and colleagues [29] also show that isoflavones reduced the rumen abundance of *Clostridium* sequences, which includes the well-studied HAB, *C*. *sticklandii* and *C*. *aminophilum* [30].

Grasses contain a variety of carbohydrates, which can be categorized as non-structural or structural. Non-structural carbohydrates in cool-season (C3) grasses include simple sugars and fructans that vary throughout the growing season [31, 32]. There are examples of fructanolytic rumen bacteria in culture, including *Butyrivibrio fibrisolvens* [33]. However, little is known about the occurrence and prevalence of the microorganisms that typically use forage fructans. Grass fructans are not currently commercially available, so the enumeration media was amended with chicory inulin, a model fructan previously employed by our research group to study fructanolytic bacteria from horses [34, 35]. When steers were grazing pasture, 10^10^–10^11^ cells mL^-1^ total fructan-utilizing bacteria were detected (Table 3). Both year (*P* < 0.01) and treatment (*P* < 0.01) effects were observed for fructan-utilizing bacteria, but there was no effect of time or any interactions between these variables (*P* > 0.05). Overall, the viable number of fructan-utilizing bacteria was higher in 2017 in comparison to 2016 (*P* < 0.05). Protein supplementation, regardless of source, decreased fructan-utilizing bacteria, with red clover hay treatments result in the most pronounced reduction (*P* < 0.05).

Cellulose is the most abundant carbohydrate on pasture, in forage, and indeed it is the most abundant biopolymer in the world [36]. Grazing ruminants have metabolic access to the calories in cellulose only through the activity of cellulolytic microbiota. Over the course of the study, 10^8^–10^11^ cells mL^-1^ total cellulolytic bacteria were enumerated with an average viable number of 3.59 × 10^9^ cells mL^-1^ detected. The viable number of total cellulolytic bacteria was unaffected by treatment (Table 3; *P* = 0.45). This result was consistent with previous *in vitro* experiments demonstrating that biochanin A did not inhibit the cellulolytic activity of rumen bacteria, even though the growth of some cellulolytic species were inhibited in pure culture [9]. In fact, *in vitro* cellulose catalysis by washed, mixed rumen microbiota was actually enhanced by 30 ppm biochanin A. The hypothesis that isoflavones could improve fiber degradation was tested in the current study using the following two *ex vivo* experiments.

### *In vitro* dry matter digestibility by cellulolytic consortia

**Fig 2:**
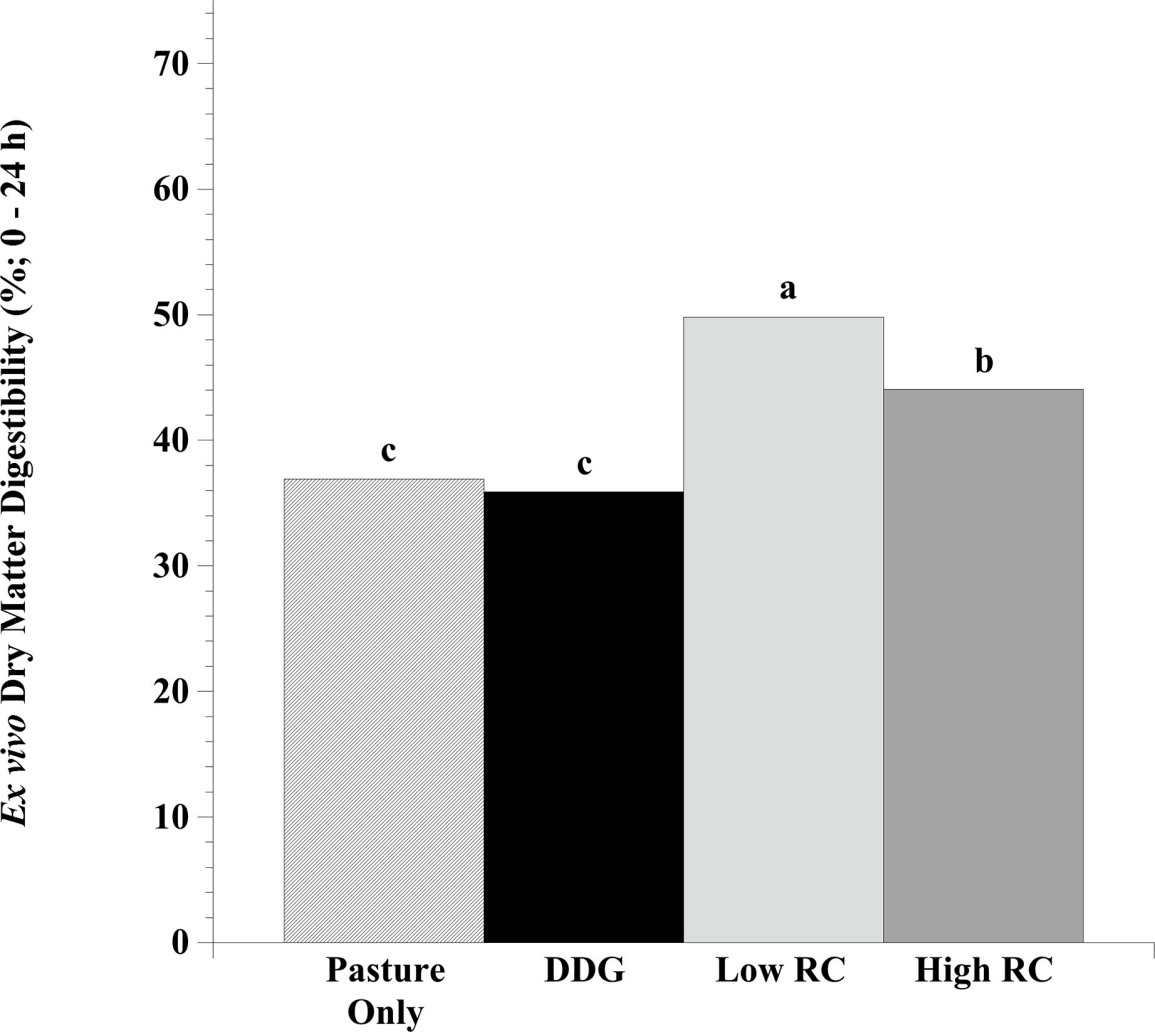
Effect of dried distiller’s grains (DDG) with or without red clover hay (RC) compared with a pasture-only control on the *ex vivo* dry matter digestibility of cellulose by predominant cellulolytic bacteria in the rumen of steers grazing a mixture of endophyte- free tall fescue (*Lolium arundinaceum*), Kentucky bluegrass (*Poa pratensis*), and orchardgrass (*Dactylis glomerata*) during the early season (9 June– 22 July, 2016; 18 May– 29 June, 2017). Ruminally cannulated Holstein steers (n = 12) were assigned to 1 of 4 treatments: Pasture only control, DDG (pasture + 2.27 kg DDG steer^-1^), Low RC (pasture + 1.51 kg DDG steer^-1^ + 0.91 kg RC steer^-1^), or High RC (pasture + 0.75 kg DDG steer^-1^ + 1.81 kg RC steer^-1^). Rumen fluid samples were collected at 4 and 6 weeks of grazing for cellulolytic bacteria enumeration. After incubation, top dilutions were evaluated for *ex vivo* dry matter digestibility of cellulose. Means lacking a common letter are different (*P* < 0.05); Year: *P* < 0.01, Treatment: *P* < 0.01, Treatment × Year: *P* < 0.01; SEM: Year = 0.40, Treatment = 0.67, Treatment × Year = 0.80.

The greatest dilution showing growth from each cellulolytic series was maintained in culture as an enriched consortium. The predominant cellulolytic consortia were subsequently incubated with hay (DM basis: 12.8% CP, 36.3% ADF, 56.3% NDF, 2.4 Mcal kg^-1^ DE). The percent *ex vivo* dry matter disappearance (% EVDMD) observed was impacted by year (*P* < 0.01), treatment (*P* < 0.01), and treatment × year (*P* < 0.01; Fig 2). Overall, the % EVDMD by predominant cellulolytic consortia was higher in 2017 than in 2016 (*P* < 0.05), but no differences were observed in treatment effects between years (*P* > 0.05). Similar % EVDMD values were observed by predominant cellulolytic bacteria from pasture-only controls and DDG supplemented steers (*P* > 0.05; Pasture-only: 37%, DDG: 36%). However, the predominant cellulolytic consortia from red clover hay supplemented steers had greater % EVDMD, with the low level of red clover hay being most effective (*P* < 0.05; Pasture only: 36%, DDG 35%, Low RC: 50%, High RC: 44%).

### *Ex vivo* fermentations of hay, corn or DDG

When rumen fluid was collected from the fistulated Holstein steers during the experiment it was used directly in digestibility experiments. Alternately, the microbial cells were harvested from the rumen fluid and washed, as described above. The difference between rumen fluid (RF) and the washed cell suspension (CS) is the rumen metabolome at the time the animal was sampled. All of the microbial products, such as short chain fatty acids and ammonia, are reduced to undetectable levels in the washing process [37]. Cell washing also removes dietary compounds, including the isoflavones and their bacterial metabolites. Isoflavones can exert effects *ex vivo* in RF because the isoflavones that the animal consumed are still present in the RF. When washed CS have an *ex vivo* effect from a dietary treatment, it is inferred that the dietary treatment altered the composition or metabolism of the microbial cells. In general, CS from red clover-supplemented steers digested hay to a greater extent than CS from pasture-only or DDG-supplemented steers (Table 4). Conversely, CS from red clover-supplemented steers digested DDG and corn to a lesser extent than CS from pasture-only or DDG-supplemented steers. These results indicate that a factor in the clover altered the microbiota that were later captured in the *ex vivo* CS. Our previous results showing the effects of biochanin A on HAB, amylolytic, and cellulolytic bacteria support the hypothesis that biochanin A was responsible for the effects by selective antimicrobial inhibition *in vivo* [6–9]. There was no treatment difference in corn fermentation when whole RF was used, but that could be because the starting SCFA inhibit fermentation to some degree.

**Table 4:** Effect of dried distiller’s grains (DDG) with or without red cover hay (RC) compared with a pasture-only control on *ex vivo* dry matter digestibility by rumen fluid (RF) or bacterial cell suspensions (CS) fermenting ground corn, hay, or DDG.

CS/RF	Treatments^1^				Statistics^2^	
	Pasture-Only	DDG	Low RC	High RC	Sig	SEM
**Corn**						
**RF**	38.9	38.6	41.2	41.3	*P* = 0.14	0.94
**CS**	49.4^a^	49.6^a^	39.8^b^	42.0^b^	*P* < 0.01	0.78
**Hay**						
**RF**	47.3^b^	33.1^c^	57.1^a^	51.9^b^	*P* < 0.01	0.71
**CS**	51.5^c^	49.6^c^	60.9^a^	56.6^b^	*P* < 0.01	1.14
**DDG**						
**RF**	41.8^a^	41.2^a^	29.6^c^	33.2^b^	*P* < 0.01	0.75
**CS**	49.0^a^	50.1^a^	39.1^b^	38.9^c^	*P* < 0.01	0.41

^1^Pasture-only: control; DDG: Pasture + 2.27 kg DDG steer^-1^; Low RC: Pasture + 1.51 kg DDG steer^-1^ + 0.91 kg RC steer^-1^; High RC: Pasture + 0.75 kg DDG steer^-1^ + 1.81 kg RC steer^-1^

^2^ Means lacking a common letter are different between treatments within year (*P* < 0.05)

Simple fermentation products, ionic hydrogen (as pH) and ammonium (as NH_3_), were quantified in *ex vivo* fermentations (Table 5). The results were consistent with the previously noted activities of biochanin A. It should be noted that differences in NH_3_ concentration were detected when DDG was the substrate, but not when corn or hay were the substrate. Many amylolytic bacteria, like *Streptococcus bovis*, assimilate NH_3_ [38]. The same is true for cellulolytic bacteria, in fact, *Fibrobacter succinogenes* was shown to lyse and die when NH_3_ was limiting [39]. Any difference in ammonia production in the hay and corn fermentations could have been offset by assimilation. Dried distillers’ grains (DDG) is compositionally different than hay or corn grain. It is rich in protein and fat; thus, it is likely that proteolytic bacteria, HAB and lipolytic bacteria would predominate in a batch fermentation with DDG as the sole substrate.

**Table 5:** Effect of dried distiller’s grains (DDG) with or without red cover hay (RC) compared with a pasture-only control on pH decline and NH_3_ accumulation in *ex vivo* fermentations of ground corn, hay or DDG by rumen fluid (RF) or bacterial cell suspensions (CS).

Δ	CS/RF	Treatments^1^				Statistics^2^	
		Pasture-Only	DDG	Low RC	High RC	Sig	SEM
	**Corn**						
**pH**	**RF**	1.44^b^	1.54^a^	1.20^c^	1.22^c^	*P* < 0.01	0.02
**NH**_**3**_	**RF**	10.99	10.67	9.03	8.02	*P* = 0.24	1.04
**pH**	**CS**	1.45	1.48	1.41	1.32	*P* = 0.06	0.02
**NH**_**3**_	**CS**	8.73	9.86	8.24	10.36	*P* = 0.11	0.56
	**Hay**						
**pH**	**RF**	0.54^c^	0.69^b^	1.06^a^	0.96^a^	*P* < 0.01	0.03
**NH**_**3**_	**RF**	15.91	12.74	13.64	11.86	*P* = 0.28	1.36
**pH**	**CS**	1.11^c^	1.11^c^	1.38^a^	1.29^b^	*P* < 0.01	0.01
**NH**_**3**_	**CS**	11.81	11.23	10.17	11.55	*P* = 0.21	0.50
	**DDG**						
**pH**	**RF**	0.66^c^	0.81^b^	1.13^a^	1.19^a^	*P* < 0.01	0.02
**NH**_**3**_	**RF**	18.46^b^	21.53^a^	13.94^d^	15.65^c^	*P* < 0.01	0.44
**pH**	**CS**	1.26^b^	1.28^b^	1.37^a^	1.38^a^	*P* < 0.01	0.01
**NH**_**3**_	**CS**	15.77^b^	22.68^a^	14.01^b^	16.20^ab^	*P* < 0.01	0.60

^1^Pasture-only: control; DDG: Pasture + 2.27 kg DDG steer^-1^; Low RC: Pasture + 1.51 kg DDG steer^-1^ + 0.91 kg RC steer^-1^; High RC: Pasture + 0.75 kg DDG steer^-1^ + 1.81 kg RC steer^-1^

^2^ Means lacking a common letter are different between treatments within year (*P* < 0.05)

## Conclusions

The initial hypotheses were that red clover hay supplementation would 1) suppress hyper ammonia-producing rumen bacteria (HAB); 2) promote cellulolytic bacteria and fiber degradation; and 3) increase average daily gain in a dose-dependent manner. The inhibition of HAB was observed in this, third *in vivo* study on the effects of isoflavones on rumen bacteria. The results were consistent with the hypothesis that the spectrum of antimicrobial activity includes the HAB that convert feed amino acids into ammonia, as demonstrated in the previous 10 years of research [5–7, 14]. The current results indicate that it was not necessary to use extracted or purified biochanin A to achieve suppression of HAB. The antibiotic-like benefit was obtained by feeding red clover hay.

A disadvantage of feed antibiotics (*e*.*g*. ionophores) is that the spectrum of activity can include the cellulolytic bacteria, and growth promotion via antimicrobial mechanisms of action is not always seen on pasture [39, 40]. Biochanin A was selectively inhibitory to pure cultures of cellulolytic bacteria but increased overall fiber digestion *in vitro* and *ex vivo* [9]. Isoflavone consumption through red clover hay neither decreased nor increased the total viable number of cellulolytic bacteria, but *ex vivo* hay digestion was enhanced in two different types of assays (+10–25% EVDMD of hay). These results indicate a net positive impact of isoflavones on the utilization of fibrous substrates in the rumen.

Previous results showed that biochanin A inhibited amylolytic bacteria, including *Streptococcus bovis* [8]. Typical forage legumes, like clovers, contain variable amounts of starch [41]. In C3 grasses, starch tends to be a smaller proportion of the nonstructural carbohydrate than fructan [42], although starch concentrations can be elevated at low temperatures [43]. *Streptococcus bovis* rapidly ferments inulin, a fructan from chicory (*Cichorium intybus*; [35]). We conjecture that *S*. *bovis* and other bacteria that are typically considered amylolytic could occupy fructanolytic niches on pasture, and that the viable number of rumen fructan-utilizers was decreased on red clover treatments. It is not clear if this latter result is beneficial for the animal. However, it is important to note that isoflavones are biotransformed in the rumen, and the metabolites are absorbed [44, 45]. Thus, there is another opportunity for fructan fermentation and SCFA absorption in the lower digestive tract where isoflavone concentrations are likely to be lower.

The third hypothesis, growth promotion, was the least clear cut. The most rapid steer growth was observed on the lower level of red clover supplementation (low RC). The greater level of red clover (high RC) was statistically no better than DDG alone, but a numerical growth benefit was observed. There are a number of nutritional factors to consider. DDG is a common supplement for ruminants, which provides protein and fat. Protein was balanced in this study with DDG because it is free of isoflavones; however, it is unclear if protein composition and quality might have differed between treatments and consequently impacted the results of the current study. Additionally, the DDG only group were supplemented with more fat than the other animals. In this case, evidence points to a factor in the red clover rather than the DDG. Fat can be inhibitory to fiber catalysis in the rumen [46]. However, the inclusion of DDG does not show a dose-dependent effect on fiber catalysis, which indicates that fat was not inhibiting fermentation.

Catalysis of the fibrous substrate hay was greatest by the low RC rumen microbiota (+10–25% EVDMD); SCFA were greatest in the rumens of low RC steers (+~25% for all SCFA); and low RC steers gained weight the most rapidly (+0.17 kg d^-1^ higher than DDG only controls). These data follow the central theory of rumen microbiology; the rumen microbiota digest what the animal cannot, and the animal uses the bacterial products for its own metabolism. It is possible that low RC supplementation simply achieved the optimal isoflavone dose for enhanced fiber utilization and suppression of HAB (approx. 1200 mg biochanin A equivalents d^-1^, 4500 mg total isoflavones d^-1^). Because biochanin A, and possibly other isoflavones, are antimicrobial, the high RC isoflavone content (approx. 2380 mg biochanin A d^-1^, 8900 mg total isoflavones d^-1^) might have exceeded minimum inhibitory concentrations of the most efficient fibrolytic bacteria. However, the possibility that there were other anti-quality factors in the hay cannot be excluded. Future research is required to evaluate growth performance benefits in cattle receiving lower levels of red clover supplementation to identify optimal supplementation strategies.

Isoflavones are mildly estrogenic, improve the blood flow of ruminants suffering from fescue toxicosis (*i*.*e*., ergotism), improve rumen fermentation in a variety of ways and promote animal growth [5–9, 47]. It is tempting to claim isoflavones are a panacea; a natural medicine, for a variety of problems faced by domestic ruminants, but there is another possible perspective. Throughout their evolutionary history, ruminants have migrated and grazed a diverse variety of plants. It is reasonable to assume that they were exposed to many plant secondary metabolites that are underrepresented in modern diets. Therefore, it should not surprise us that plant secondary metabolites, like isoflavones, play undiscovered roles in ruminant nutrition.
